# Supplementary material for: Type I IFN stimulates lymph node stromal cells from adult and old mice during a West Nile virus infection
Source: Aging Cell. 2023 Feb 17;22(4):e13796. doi: 10.1111/acel.13796 (PMC10086524; doi:10.1111/acel.13796)
Supplement: Supplementary file 1 — Figure S1 [file ACEL-22-e13796-s002.pdf]

Type I IFN stimulates lymph node stromal cells from adult and old mice during a West Nile virus infection

Allison K. Bennett[1], Michelle Richner[1], Madeline D. Mun[1], Justin M. Richner[1]#

[1]Department of Microbiology and Immunology, University of Illinois College of Medicine, Chicago, IL, USA

Supplemental Figure Legends

Supplementary Figure 1: WNV infection triggers expansion of LNSC subsets in adult and aged DLNs *in vivo*
Adult (8-10 weeks) and old (18 months) C57BL/6J mice were subcutaneously infected with $2x10^3$ FFU of WNV-Kunjin. Draining popliteal LNs were harvested from infected mice at days 2,4,6, and 10 after infection. At each timepoint, frequencies of proliferating Ki67+ FRCs (**A**), LECs (**B**), and (**C**) BECs were quantified in adult and old DLNs. The results are averaged from 2 independent experiments with 3 mice per timepoint. Data is expressed as the mean ± SEM. Statistically significant differences between adult and old groups at each timepoint is denoted by asterisks (*, $P < 0.05$; ***, $P < 0.001$; Multiple unpaired T tests).

Supplementary Figure 2: Characterization of *ex vivo* LNSC culture systems
LNSCs from skin draining LNs from adult C57BL/6J mice were digested and expanded in gelatin-coated dishes with (**A**) αMEM media or (**B**) endothelial cell medium. Growth of major LNSC subsets was confirmed by flow cytometry. (**A,B**) Representative dot plots show the gating strategy used to identify LNSC subsets in each culture system. LNSCs (gated on $CD45^-$ cells) were divided into LEC (LYVE-1^+) and non-LEC (LYVE-1^-) populations based on Pdpn and LYVE-1 staining (left). The LYVE-1^- population was divided into FRCs and BECs based on Pdpn and CD31 staining (right). (**C**) Representative images of *ex vivo* LNSC cultures grown in the presence of αMEM media (top) or endothelial cell medium (bottom).

Supplementary Figure 3: BMDC-WNV supernatants contain both inflammatory cytokines and infectious virus
Bone marrow-derived dendritic cells (BMDCs) were infected for 20 hours with WNV-Kunjin virus (MOI=10), and supernatants were collected for further characterization. Infectious WNV-Kunjin titers were quantified by FFA. Secreted levels of IFNα and chemokines were quantified by ELISA and 31-Plex Multiplex assay, respectively.

Supplementary Figure 4: LNSC activation is not augmented by increasing poly I:C concentration
LNSCs cultured in endothelial cell media were incubated with increasing concentrations of poly I:C (125 ng/ml-1μg/mL) for 20 hours. *Cxcl9* expression was quantified by qRT-PCR and normalized to untreated. Data is expressed as the mean ± SEM.

Supplementary Figure 5: Age does not significantly drive global gene expression variability in LNSCs
Principal component analysis (PCA) was performed on normalized RNA-seq data of adult and old LNSCs from *ex vivo* stimulation experiments: untreated, BMDC-WNV, and rIFNα. Each dot represents a single sample and is colored based on sample type. Adult and old samples cluster together based on treatment, rather than age.

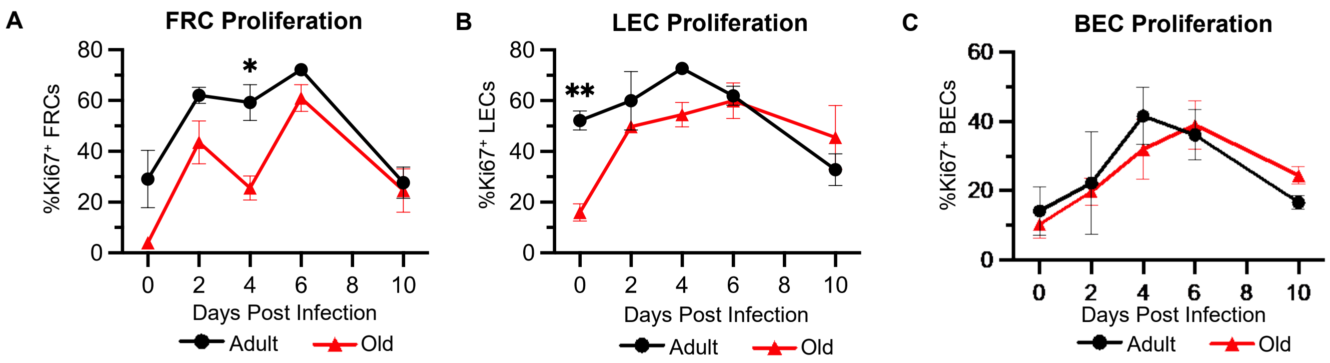

Supplemental Figure 1

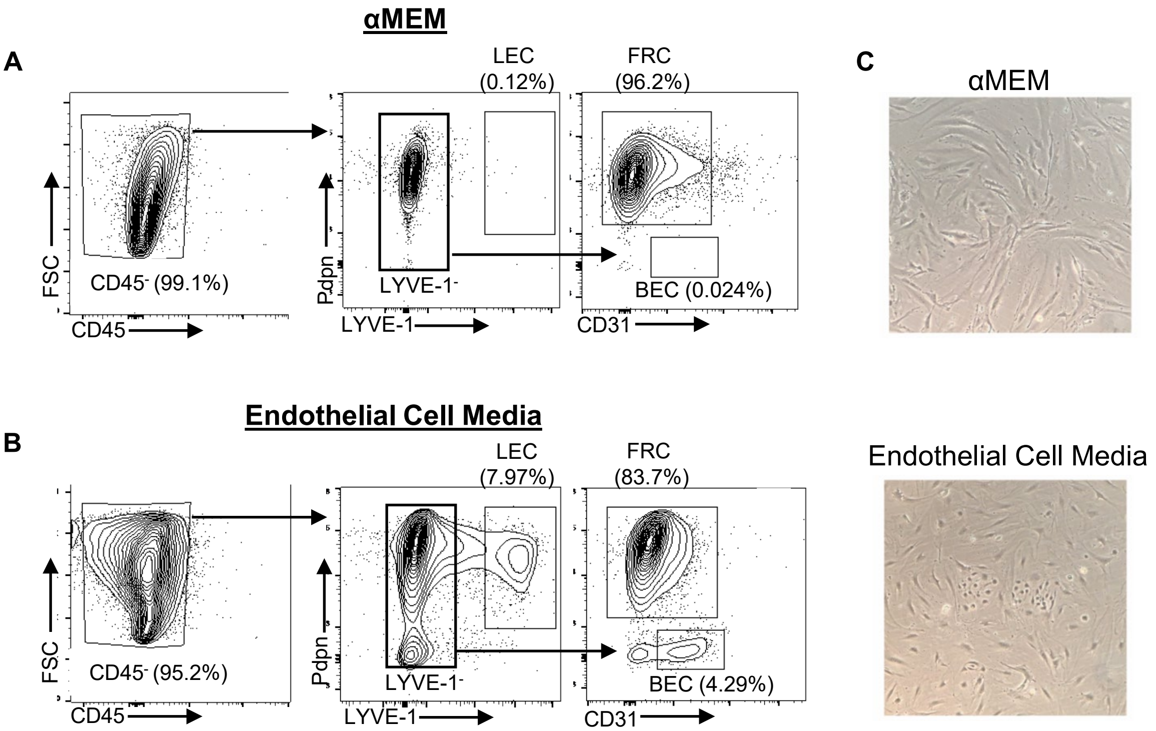

Supplemental Figure 2

Infectious Virus	(FFU/mL)
WNV-Kunjin	$6.63 \times 10^5 \pm .59 \times 10^5$
**Cytokines**	**(pg/mL)**
IFNα	85 ± 38
CCL20	5 ± 3
IL-1β	12 ± 5
CXCL5	132 ± 48
TNF-α	87 ± 91
GM-CSF	0.62 ± 0.14
CX3CL1	570 ± 1209
CCL1	1 ± 0.5
CXCL13	16 ± 11
IL-16	10 ± 3
IFNɣ	5 ± 0.8
CXCL10	61682 ± 40584
IL-2	0.95 ± 0.11
CXCL11	361 ± 206
IL-6	14 ± 7
IL-4	12 ± 12
CCL7	24 ± 12
CCL12	65 ± 50
CCL2	473 ± 294
CCL22	840 ± 222
CCL5	122 ± 58
IL-10	476 ± 368
CXCL1	44 ± 21
CCL27	296 ± 229
CCL17	1897 ± 493
CCL19	677 ± 444
CXCL16	125 ± 24
CXCL12	211 ± 91
CCL24	194 ± 104
CCL11	0.74 ± 0.42
CCL4	411 ± 159
CCL3	27 ± 10

Supplemental Figure 3

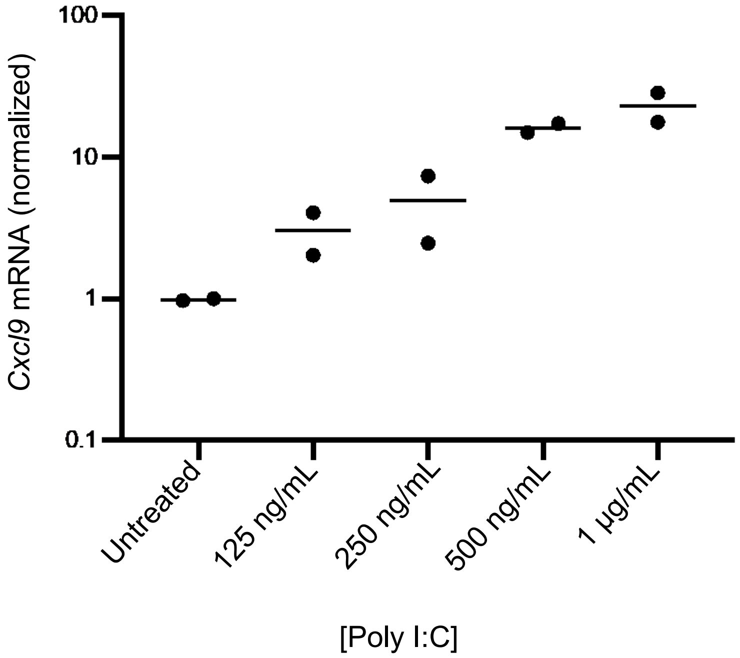

Supplemental Figure 4

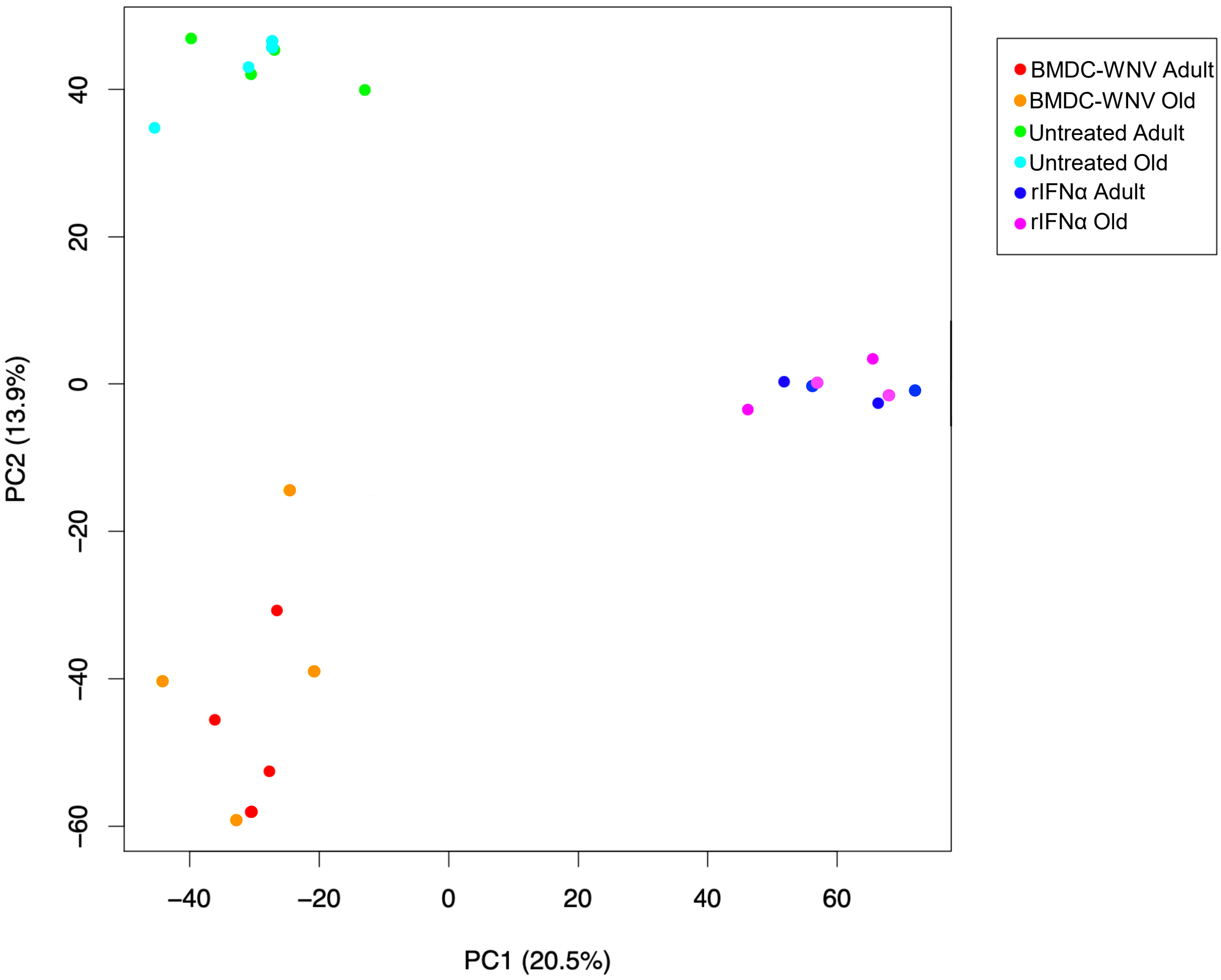

Supplemental Figure 5
